# Activated Nickel Foam Anodes for Sustainable Biomass Valorization: Competitive Oxidation of Organic Molecules vs the Oxygen Evolution

**DOI:** 10.1021/acs.energyfuels.5c05778

**Published:** 2026-02-16

**Authors:** Rudy Crisafulli, I. Rafael Garduño-Ibarra, Sravan K. Kilaparthi, Paula Sánchez, Antonio de Lucas-Consuegra

**Affiliations:** † Department of Chemical Engineering, Faculty of Chemical Sciences and Technologies, 16733 University of Castilla-La Mancha, Ciudad Real 13005, Spain; ‡ Instituto de Pesquisas Energéticas e Nucleares, IPEN/CNEN-SP, Av. Professor Lineu Prestes 2242 CEP 05508-000, São Paulo, SP, Brazil

## Abstract

A systematic study on the competitive oxidation of glucose (Glc), xylose (Xyl), and 5-hydroxy­methyl­fur­fural (HMF) vs the oxygen evolution reaction (OER) was performed by coupling H-cell electrochemical experiments with *in situ* O_2_ monitoring in the anodic chamber using an activated Ni foam as the anode. At a substrate concentration of 10 mM, multipotential steps showed similar OER onset potential values for Glc and Xyl (1.49 V_RHE_), while the value for HMF was slightly lower (1.47 V_RHE_). Chronoamperometry tests at 1.6 V_RHE_ (30 min) with varying concentrations showed that both Glc and Xyl oxidation reactions fully suppressed the OER at 30 mM, while 100 mM was required for HMF. A Langmuir–Hinshelwood analysis of the current–substrate concentration dependence revealed the slower kinetics and inhibitory effects impacting HMF oxidation, which account for the significant difference in performance with respect to both aldoses. Given its relevance as both a model and a promising substrate for membraneless electrolysis operation, Glc was further investigated in a long-term chronoamperometry experiment with *in situ* O_2_ monitoring (15 h at 1.6 V_RHE_, 30 mM Glc). The results suggested the feasibility of sustaining OER-free operational conditions for approximately 4 h from an initial Glc concentration of 100 mM. HPLC analysis indicated the presence of formate as the main coproduct of hydrogen via glucose electrolysis.

## Graphical abstract

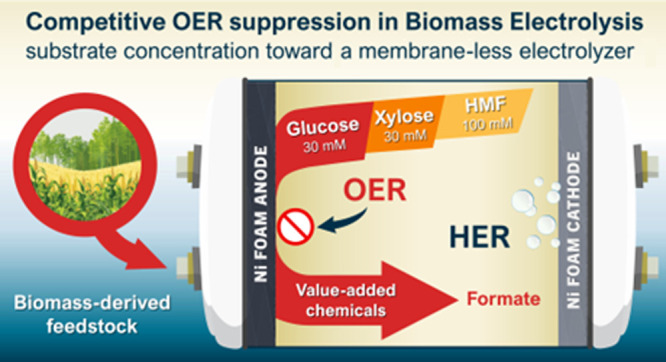

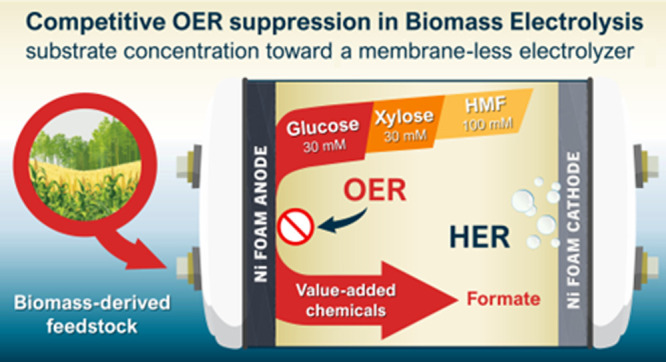

## 1 Introduction

Green hydrogen production via alkaline water electrolysis (AWE) is a strategic avenue to decarbonize industrial and energy sectors, an essential step toward a sustainable, carbon-free society.^1^ However, the oxygen evolution reaction (OER) taking place at the anode is a thermodynamically demanding and kinetically sluggish reaction, often requiring high overpotentials and yielding only O_2_ as a low-value byproduct.^2^ A promising strategy widely investigated in recent years is to overcome these limitations by replacing the OER with another electrochemical reaction, namely, biomass-derived molecules electro-oxidation. This approach not only decreases the cell voltage for hydrogen production due to favorable kinetics and thermodynamics but also enables the coproduction of value-added chemicals at the anode.^3,4^ Among the most studied non-noble metals for such purposes, Ni-based materials stand out on account of the redox couple Ni^2+^/Ni^3+^, or Ni­(OH)_2_/NiOOH species, where NiOOH is identified not only as the catalytically active phase in OER^5,6^ but also in the oxidation of organic substrates.^7^

Nickel foam (NF) is an inexpensive commercial material widely used due to its electrical conductivity, high specific surface area, and good structural stability, especially under the corrosive environment of AWE.^8,9^ The high-valence Ni species can be usually grown in the surface of NF through electrochemical activations, in which, with applied anodic potentials, the surface undergoes the enrichment of the Ni^2+^/Ni^3+^ species.^6,10^ In general, the oxidation of organic compounds is mediated by the Ni^2+^/Ni^3+^ redox couple, where the reduction of Ni^3+^ back to Ni^2+^ is coupled to a proton transfer from the oxidized substrate, a mechanism referred to as the Fleischmann mechanism.^11^ A potential-dependent mechanism implies the Ni^3+^ at higher potentials is the active surface for the oxidation of the adsorbed substrate.^7^ In this case, substrates are first adsorbed on the negatively charged Ni^3+^ surface, and the oxidation proceeds until the product is formed and desorbed by deprotonation, fully reducing Ni^3+^ back to Ni^2+^. The applied potential thus reoxidizes Ni^2+^, regenerating the consumed Ni^3+^ active sites.^12^ In this mechanism, however, there is competition for the active sites in the surface between the organic substates and OER species, (i.e., *O, *OH, *OOH).^13^ Hence, the OER constitutes a key limiting factor in organic electro-oxidation reactions. A recent study reported the impact of substrate concentration (ethanol) and the operating conditions with respect to glucose electro-oxidation reaction (GOR) catalyzed with Ni^2+^/Ni^3+^ and its competition with OER.^14^ In this vein, a recent work by our group demonstrated that under mild alkaline conditions (0.1 M NaOH + 1.0 M Na_2_SO_4_, pH < 13), glucose (Glc) concentrations above 40 mM are sufficient to fully suppress OER during anodic polarization at 1.7 V vs RHE, using a commercial Ni catalyst (20 wt % Ni on Vulcan carbon).^15^ From these results, it was proposed that under such conditions a membraneless electrolyzer (MLE) oxidizing Glc could be operative.

However, GOR and OER are usually studied at stronger alkaline conditions (typically at ≥1.0 M KOH or NaOH). Following this path, the present work reports the potential threshold at which Glc can be oxidized without concomitant O_2_ production under a more alkaline environment (1.0 M KOH). In addition, in this work the anode material employed was a commercial NF, given its good structural stability under strong basic conditions, as opposed to carbon-supported catalysts which are prone to suffer alkaline corrosion.^16^ Additional tests were performed by using two other biomass-derived molecules of commercial interest. The first was xylose (Xyl), from which valuable organic acids (e.g., xylonic acid) can be obtained.^17^ The second was 5-hydroxy­methyl­fur­fural (HMF), whose complete oxidation yields 2,5-furan­dicarb­oxylic acid (FDCA), a highly appreciated commodity in the bioplastics industry.^18^ Xylose electro-oxidation reaction (XOR) has been considerably less studied than GOR, and mostly with noble-metal-based catalysts.^19^ In contrast, HMF electro-oxidation reaction (HMFOR) has received increasing attention in recent years, although much remains to be understood regarding OER competition.^20^ Despite the abundance of Xyl in biomass, the concentration threshold beyond which XOR dominates over the OER has received considerably less attention than GOR. This is particularly evident for Ni-based electrocatalysts. Recent studies have reported the inhibitory effect of XOR over OER, although the optimal concentration has not been systematically established.^21,22^

Considering the above, the aim of the present work is to perform a systematic study on the competitive GOR, XOR, and HMFOR with the OER. For that reason, a commercially available Ni foam was employed instead of a synthesized catalyst to ensure high reproducibility and reliability of the experimental methodology, particularly for the combined electrochemical and *in situ* O_2_ sensing measurements. Hence, three main novelties were introduced vs previous related studies: (i) Comparison between three different organic molecules (glucose, xylose, and HMF) working under 1 M NaOH electrolyte conditions. (ii) A commercial Ni foam was used instead of a spray coated Ni/C catalyst. (iii) For the proof of concept, a long-term CA experiment at 1.6 V vs RHE with 100 mM Glc was carried out for 15 h, with simultaneous HPLC analysis during membraneless operation regime. Potentials were varied from 1.43 to 1.55 V vs RHE under steady-state current conditions, while the O_2_ evolution was measured *in situ* in the headspace of the anodic chamber of a tightly sealed H-cell. It should be emphasized that the membraneless operation discussed in this work is conceptual, as a true single-chamber cell was not implemented. Instead, a divided H-cell equipped with an anion-exchange membrane was intentionally used to prevent cathodically generated hydrogen from diluting or interfering with the atomic density of the O_2_ sensor. Within this configuration, our results allow the identification of the substrate, concentration, and potential conditions under which OER can be fully suppressed, thereby providing a solid foundation for the future development of practical membrane-free electrolyzer systems. This study aligns with the objectives of the ELOBIO European Project (Electrolysis of Biomass), which focuses on the development of MLE for the simultaneous production of hydrogen and value-added chemicals.

## 2 Materials and Methods

### 2.1 Chemicals

The working electrode (WE) material was an NF with 93% porosity and 0.9 mm thickness (Goodfellow Materials). For the studied substrates, (Glc, Xyl, and HMF), d-(+)-glucose anhydrous 99% (Thermo Scientific), d-(+)-xylose ≥ 99% (Sigma-Aldrich), and 5-hydroxy­methyl­fur­fural ≥99% (Sigma-Aldrich) were used. HMF was stocked at 4 °C to minimize degradation and exposure to air. Pure NaOH and 85% KOH (AppliChem) were used for the alkaline solutions. For the HPLC standards, in addition to glucose, formic acid ≥99% (Sigma-Aldrich) and oxalic acid anhydrous 98% (Thermo Scientific) were used.

### 2.2 NF Activation and Electrochemical Tests

NF activation and electrochemical measurements (which included cyclic voltammetry (CV), linear sweep voltammetry (LSV), chronoamperometry (CA), and electrochemical impedance spectroscopy (EIS)), were performed in a tightly sealed H-cell with a 75 mL working volume in each chamber (DEK Research), controlled by an OrigaLys OGF500 potentiostat. The reference electrode (RE) was Hg/HgO (1 M NaOH) (Corrtest Instruments) with a Luggin capillary, and a bare NF was placed in the cathode chamber as a counter electrode (CE), with dimensions of 20 mm × 20 mm × 2 mm. The anion-exchange membrane (AEM) used to separate the chambers was a Sustainion X37-50 membrane, which was pretreated by immersing it in a 1.0 M KOH solution for at least 24 h. All tests were conducted at 25 °C, and all potentials are reported vs RHE using the expression

1 $$E_{\mathrm{RHE}}=E_{\mathrm{Hg}/\mathrm{HgO}}+0.059\times \mathrm{pH}+E_{\mathrm{Hg}/\mathrm{HgO}}^{0}$$

where *E*_Hg/HgO_ is the WE potential measured at a given current, and E_Hg/HgO_^0^ is the standard potential of the reference electrode after calibration, 0.098 V. From this point onward, all potentials are expressed in relation to the RHE.

To prepare the electrode, first, the NF was cut into pieces to obtain a submergible area of 10 mm × 10 mm × 2 mm (geometric electrode area). Then, the pieces were pretreated by an ultrasonic bath in 3.0 M HCl for 30 min, followed by a thorough rinse and 5 min ultrasonic bath in ultrapure water. To minimize capillary effects, the NF was subjected to a hydraulic press of 1 ton for 15 min. Later, CV was performed to activate the Ni foam scanning from 0.0 to 1.55 V at 100 mV s^–1^ in 1.0 M NaOH until the current was stable (800–1000 cycles). The ohmic resistance of the electrolyte (*R*_e_) was found to be 1.48 Ω, determined by the Nyquist plot at open-circuit potential (EIS performed between 100 mHz and 1 kHz, 10 mV amplitude). Electrochemical impedance spectroscopy was employed primarily as a diagnostic tool to determine the solution resistance and ensure stable cell operation over time, allowing accurate *iR* compensation of the applied potentials. The measured resistance remained essentially constant (∼1.3–1.4 Ω) before and after Ni foam activation, confirming the reproducibility of the electrochemical setup.

To determine the O_2_ onset potentials, a series of 5 min CA studies were performed between 1.43 and 1.55 V, with increments of 0.01 V. The concentration of each substrate in these preliminary tests was 10 mM. The following experiments were 30 min CA studies conducted at a fixed potential (1.6 V), first without substrate and then varying the substrate concentration from 10 to 50 mM in the case of Glc and Xyl and up to 100 mM in the case of HMF. The O_2_ production was monitored during CA in the gas phase in the headspace of the anodic chamber using a PyroScience OXSP5-ADH optical sensor fixed inside the anodic chamber. The *in situ* O_2_ measurements of each test enabled the estimation of the O_2_ faradaic efficiency (FE_O_2__) as follows:

2 $${\mathrm{FE}}_{O_{2}}\left[\%\right]=100\times \frac{n_{O_{2},\exp}}{n_{O_{2},\mathrm{far}}},\mathrm{with}\;n_{O_{2},\mathrm{far}}=\frac{Q}{zF}$$

where *n*_O_2_,far_ is the FE_O_2__, *n*_O_2_,exp_ is the molar mass of O_2_ measured experimentally, *Q* is the total charge passed during the experiment, *F* is the faradaic constant 96,485.6 C mol^–1^, and *z* is the number of electrons (*z* = 4) for the OER in the alkaline medium

$$4{\mathrm{OH}}^{-}(\mathrm{aq})\to O_{2}(g)+2H_{2}O(l)+4e^{-}$$ 3

To ensure O_2_-free conditions, the anode and cathode chambers were purged with a N_2_ flow before each experiment, at open-circuit potential, for a period of 30 min. During the electrochemical experiments, the N_2_ flow from the anode chamber was stopped to prevent O_2_ being flushed out of the anodic chamber, thus ensuring reliable detection of even the smallest changes in O_2_ concentration.

### 2.3 Material Characterization

Scanning electron microscopy (SEM) images were obtained using a ZEISS Gemini SEM 500 FE-SEM with a PIN-diode BSE detector. To determine the elemental composition, energy-dispersive X-ray spectroscopy (EDX) from Oxford Instruments was employed. X-ray photoelectron spectroscopy (XPS) was carried out to assess chemical and electronic configurations with a PHI VersaProbe II spectrometer from Physical Electronics operating at 49.1 W with monochromatic Al Kα radiation (*h*υ = 1486.6 eV). High-resolution spectra were recorded over an analysis area that was 200 μm in diameter by using a constant pass energy mode at 29.35 eV.

### 2.4 Product Analysis

The main electro-oxidation liquid products were determined only for GOR, which was conducted in a 15 h CA test at 1.6 V, at the minimum concentration where the OER was suppressed. Samples were taken every hour and analyzed in a Jasco LC-Net II/ADC HPLC, using a Flavor Green H 300 mm × 8 mm column and with two detectors, a UV–vis detector (Jasco UV-2075) at 210 nm and refractive index detector (Waters 2410). The column was kept at 60 °C. The mobile phase was 7 mmol of H_2_SO_4_ eluted at 0.6 mL min^–1^. The elution time of each sample was 20 min, and the injection volume was 20 μL. Before the injection, the samples were neutralized with 0.1 M H_2_SO_4_ solution.

## 3 Results and Discussion

### 3.1 Nickel Foam Activation and Characterization

The competition between OER and GOR was investigated in a conventional H-type cell separated by an AEM, with an embedded optical sensor used to monitor O_2_ production during electro-oxidation (Figure 1a). Prior to these measurements, the NF was electrochemically activated, and its morphological changes were analyzed. A marked increase in the electrochemical activity was observed during the initial cycles, particularly at the Ni^2+^/Ni^3+^ transition peaks in both the forward and reverse scans (Figure 1b). A shoulder also appeared in the reverse scan between 1.15 and 1.25 V, which grew and shifted gradually to lower potentials, which can be attributed to the presence of γ-NiOOH, evolved due to the overcharging of β-NiOOH upon cycling.^10,23^ Once the current was stable, the Ni^2+^/Ni^3+^ oxidation and reduction peaks were found at around 1.39–1.40 and 1.29–1.30 V, respectively (*E* – *iR*_e_), in agreement with the reported literature.^24,25^ The initial and final *R*_e_ measured by EIS were 1.4 and 1.3 Ω, respectively. The increasing activity of the NiOOH species accounted for an enhanced electrochemically active surface area (ECSA), which is attributed to both surface and morphological transformations induced by the activation treatment.^26^ The first noticeable change observed is the darkened NF surface (Figure 1c,f), which suggests the formation of high-valence Ni species.^27^ This was confirmed by the EDX analysis as seen in Figure 1e,h (further details in Figure S1 in the Supporting Information), in which a higher Ni oxidation state is evidenced by the adsorption of more O atoms in the activated NF surface compared to the untreated NF.

**Figure 1:**
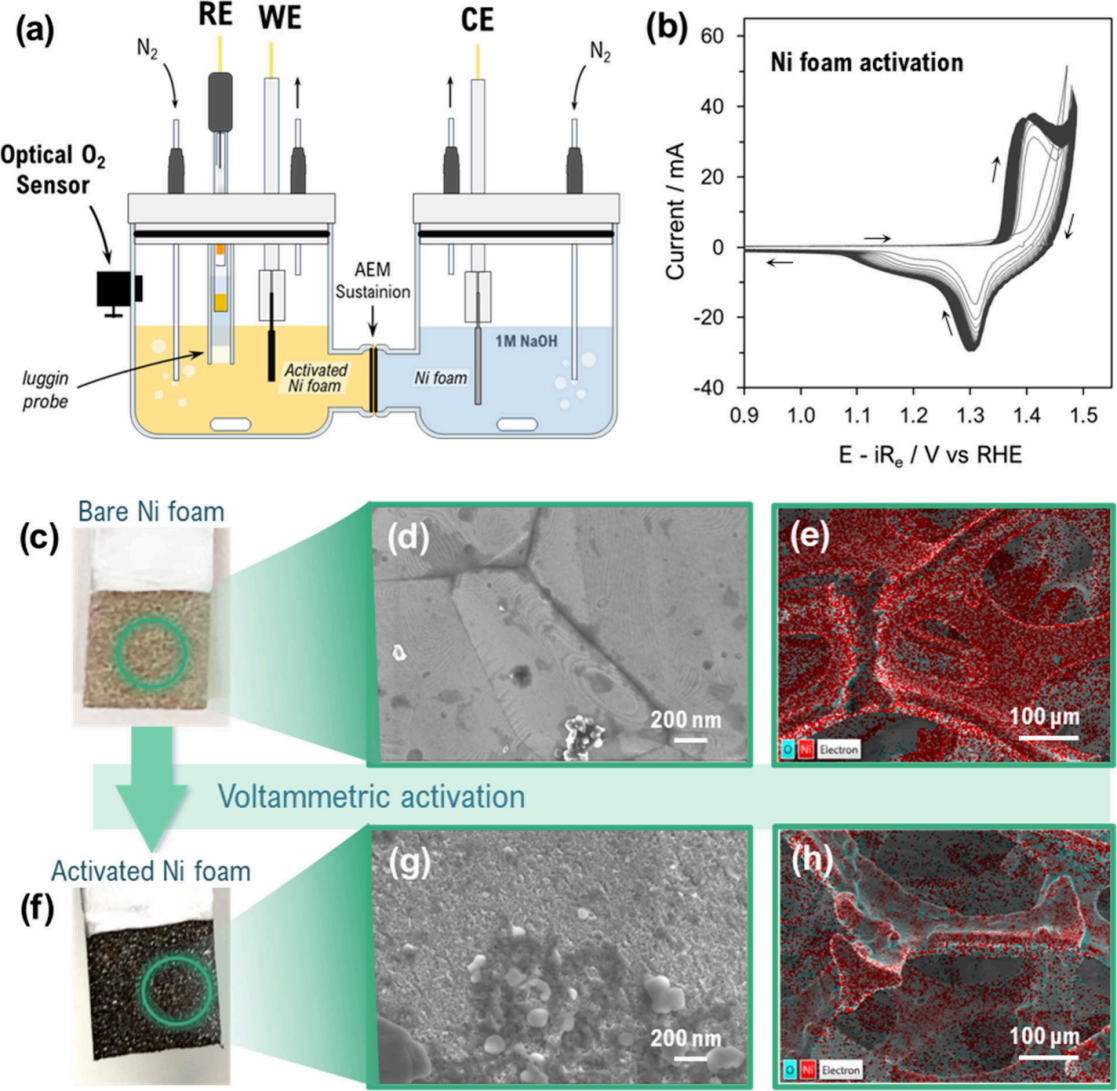
Ni foam activation process. (a) Electrochemical cell scheme used for Ni foam activation and chronoamperometric tests with *in situ* optical O_2_ detection. (b) Cyclic voltammograms recorded during activation (*iR*_e_-corrected, 100 mV s^–1^). Optical images of Ni foam (c) before and (f) after activation, with corresponding (d, g) SEM images and (e, h) EDX mapping, showing the oxygen distribution in cyan associated with Ni­(OH)_2_/NiOOH formation.

FE-SEM analysis was performed to study the morphological changes associated with the activation treatment (Figure 1d,g), in which the NF surface before and after activation reveals an enhanced surface roughness and its consequent increase in porosity as a result of the electrochemical treatment. These structural modifications favor better electrolyte penetration due to the exposed additional active sites, contributing to the enhancement of the ECSA.^28^ The structural modifications observed are not only directly related with enhanced OER activity. The development of an active Ni­(OH)_2_/NiOOH-enriched surface on the NF is essential for enabling organic electro-oxidation. To further verify the activation of the Ni foam, XPS analysis was performed on bare and activated Ni foam (Figure S2). The high-resolution Ni 2p spectrum of the bare Ni foam was deconvoluted into multiple components corresponding to Ni^0^, Ni^2+^, and Ni^3+^, with characteristic peaks located at approximately 852.6, 854.1, and 856.1, respectively, in good agreement with literature reports.^24^ Similarly, the O 1s XPS analysis revealed contributions associated with NiO and NiO_*x*_(OH)_*y*_ species, appearing at binding energies around 529.6 and 531.7 eV, consistent with the oxidized nickel species observed in the Ni 2p region. An additional peak at approximately 533.3 eV is attributed to carbonyl species.^24^

In contrast, the XPS spectra of the activated Ni foam showed clear differences. The Ni 2p spectrum is dominated by oxidized nickel species, with a prominent contribution from NiO_*x*_(OH)_*y*_ centered around 855.8 eV and the disappearance of the metallic Ni^0^ peak, indicating successful activation of the Ni foam.^24^ Furthermore, the O 1s spectrum of the activated sample exhibits an increased contribution from oxygen-containing species, further confirming the formation of an oxidized nickel surface upon activation.

### 3.2 Glucose Oxidation

After NF activation, the electro-oxidation experiments were first performed in the presence and absence of Glc. The LSVs in Figure 2a show that in the presence of 10 mM Glc, a higher electrocatalytic activity and lower onset potential were observed, showing the more favorable kinetics and thermodynamics of GOR vs OER. Moreover, GOR also starts at a much lower potential (∼1.2 V) than Ni^2+^ oxidation. It has been argued that the latter could be interpreted as evidence that GOR can occur without the mediation of NiOOH.^29^ Recent studies suggest that Glc is chemisorbed on Ni­(OH)_2_ sites before being oxidized by NiOOH at higher potentials (above ∼1.4 V).^30^ However, the FTIR spectra evidence of GOR on a polycrystalline Ni disk also suggests that the Ni^2+^/Ni^3+^ transformations may occur well below the apparent onset in the presence of Glc, that is around 1.17 V.^31^ At much higher potentials, however, the presence of OER intermediates (i.e., *OH, *O, *OOH) may compete for the active sites or even promote nonfaradaic reactions, thus affecting the selectivity of certain byproducts, (i.e., glucaric acid) or simply lowering the oxidation rate of Glc and its derivatives.^29,32^

**Figure 2:**
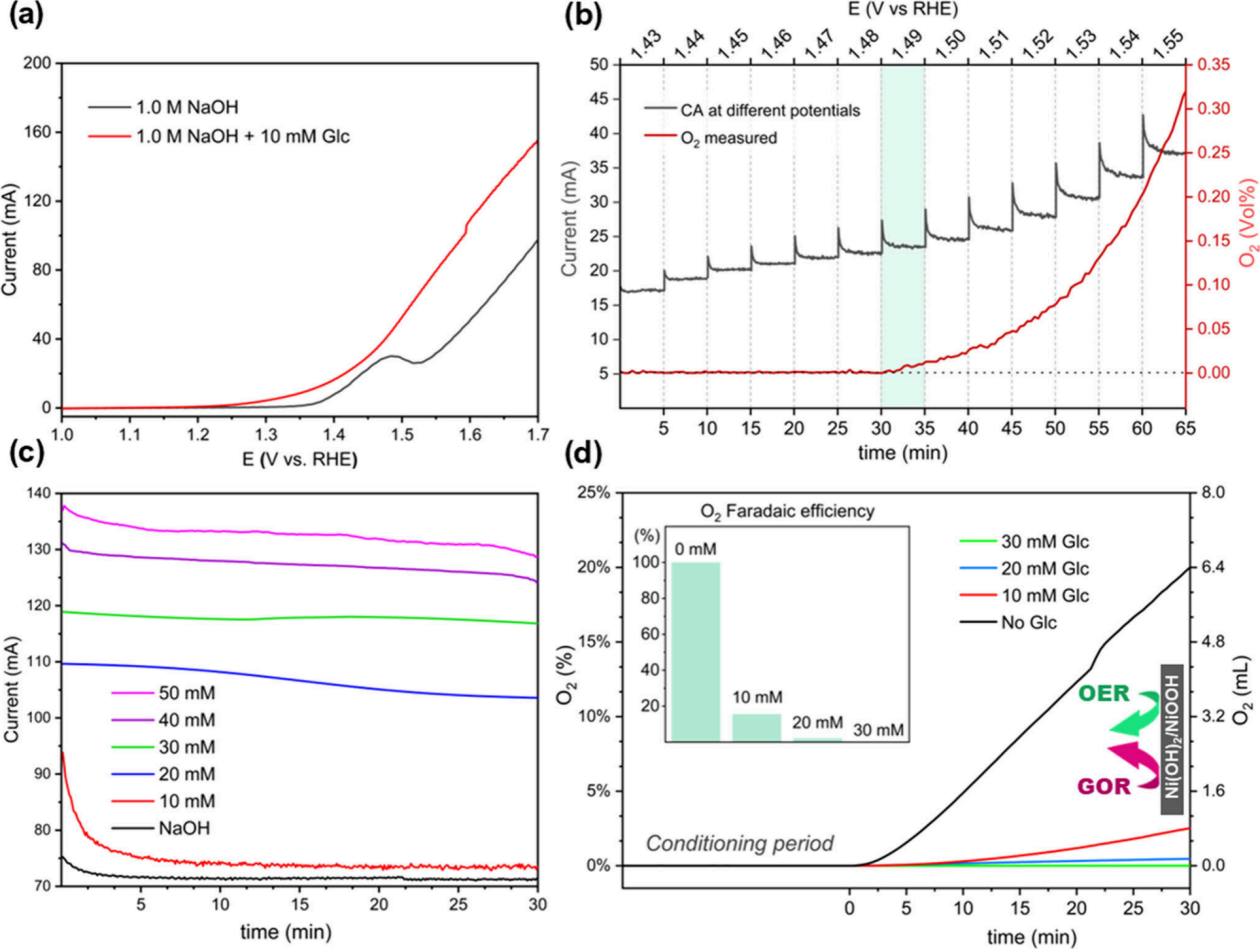
Glucose electro-oxidation on activated Ni foam. (a) LSV curves (5 mV s^–1^) in 1.0 M NaOH with and without 10 mM Glc. (b) Multistep chronoamperometry with *in situ* O_2_ detection at 10 mM Glc to determine the O_2_ onset potential. (c) Chronoamperometric profiles at different Glc concentrations (30 min). (d) O_2_ evolution during the 30 min tests and the corresponding faradaic efficiency of the corresponding aqueous solution of the O_2_ (FE_O_2__) showing clear OER suppression at 30 mM Glc.

Since both GOR and OER can occur simultaneously at potentials above 1.4 V, voltammetric techniques such as LSV can present operational difficulties in accurately identifying the potential at which O_2_ evolution begins.^15^ However, CA study coupled with *in situ* O_2_ detection, as proposed here, provides a more reliable approach for accurately determining the actual OER onset potentials by continuously measuring the slightest change in O_2_ concentration in the headspace of the anodic chamber with high precision (detection limit of 0.02%). By gradually increasing the potential from 1.43 to 1.55 V in 5 min intervals, the measurements revealed the real OER onset at 10 mM Glc in 1.0 M NaOH, which occurs at 1.49 V (Figure 2b). Therefore, this represents the minimum potential at which O_2_ evolution was experimentally detected and, thus, the actual OER onset potential under these conditions. The next step was to determine the Glc concentration at which OER suppression occurs at a given potential, shown further below.

In optimal operating conditions, conventional alkaline electrolyzers easily achieve cell potentials of around 1.8–1.9 V. At these potentials, GOR on Ni-based catalysts yields gluconic acid with high selectivity.^29^ However, in membraneless systems, potentials above 1.6 V can trigger a vigorous hydrogen evolution reaction (HER), affecting the overall efficiency.^22^ Glc oxidation, therefore, is usually studied at a potential range between 1.4 and 1.6 V, where formate is often reported as a major byproduct within this potential range.^31,33^ Moreover, although GOR and OER still occur simultaneously at 1.6 V, the promoting effect of Glc concentration on the reaction kinetics at the electrode surface of Ni-based catalysts becomes more pronounced at this potential, as demonstrated through EIS by Wang et al.^34^ Bearing all this in mind, a further CA assessment coupled with *in situ* O_2_ detection was performed for a longer period (30 min) at a constant potential of 1.6 V while the glucose concentration was varied. As expected, higher concentrations resulted in higher current densities due to the increase in the kinetics with respect to the organic concentration (Figure 2c). At 10 mM Glc, a sudden drop is observed after 5 min due to the Glc consumption, followed by a stable behavior similar to the test without Glc, both with final currents differing by only a few mA (2–4 mA). As the Glc concentration increased, particularly at 30 mM, the initial current value was not only significantly higher, but the current all along the experiment exhibited a steady behavior, whereas at 20 mM a slight decrease in current was observed after 10 min of electrolysis. The stability can be associated with the suppression of OER. The O_2_ volume produced during each 30 min experiment is depicted in Figure 2d. In the absence of Glc, the generation of O_2_ followed a linear trend from the beginning, reaching 6.4 mL. The accuracy of the *in situ* measurement is supported by the estimated 98% FE_O_2__ in this test. In correspondence with the current recorded at 10 mM Glc, O_2_ production began after 5 min with 30% FE_O_2__, attaining a cumulative volume of 1.10 mL. At 20 mM Glc, a slight increase in O_2_ signal was observed after 10 min, attaining a cumulative volume of 0.15 mL and 5% FE_O_2__. At 30 mM Glc, no O_2_ was detected at all, confirming the complete suppression of the OER at these conditions. The latter confirms that as the Glc concentration increases, OER is suppressed by the competition with the organic electro-oxidation reaction.

### 3.3 Oxidation Competition Study between OER and GOR, XOR, and HMFOR

The same screening methods shown above for GOR were repeated for the other two proposed molecules, HMF and Xyl (Figures S3 and S4 in the Supporting Information). To ensure reproducibility, each time the used working electrode was pretreated in HCl and followed the same activation procedure, as explained in Section 2.2, until a reproducible reference behavior was attained. Additionally, given the differences in catalyst formulation, electrolyte composition, and cell configuration among reported studies, literature comparisons in this work are discussed in a qualitative context.

To describe the competitive adsorption between OH* and the organic substrates, we adapted the Langmuir–Hinshelwood (L–H) mechanism, which considers the bimolecular surface reaction between adsorbed OH* and the substrate (*θ*_OH*_·*θ*_S_) as the rate-determining interaction. Following previous reports,^35^ the experimental current densities as a function of concentration were fitted with isotherms derived from the Temkin model, according to Tian et al.,^35^ in which the substrate surface coverage is proportional to log­(*C*) in the low-to-intermediate range (Supporting Information). As seen in Figure 3, the experimental average current densities as a function of substrate concentration from the 30 min CA tests were fitted, posing the potential at 1.6 V, where the L–H mechanism is dominant.^20,30^ This approach reveals that Glc and Xyl share consistent adsorption strengths (*K* ≈ 115–120 M^–1^), as evidenced by the steep current increase at low concentrations. Yet Glc sustains a larger *j*_max_ (∼139 vs 120 mA cm^–2^ for Xyl), and the model predicts a more stable current with increasing concentration, confirming its stronger OER suppression. Xyl, in turn, reaches a maximum current at 40 mM, after which the current sharply declines. This contrast stems from differences in substrate stability in alkaline media and the associated risk of electrode poisoning.

**Figure 3:**
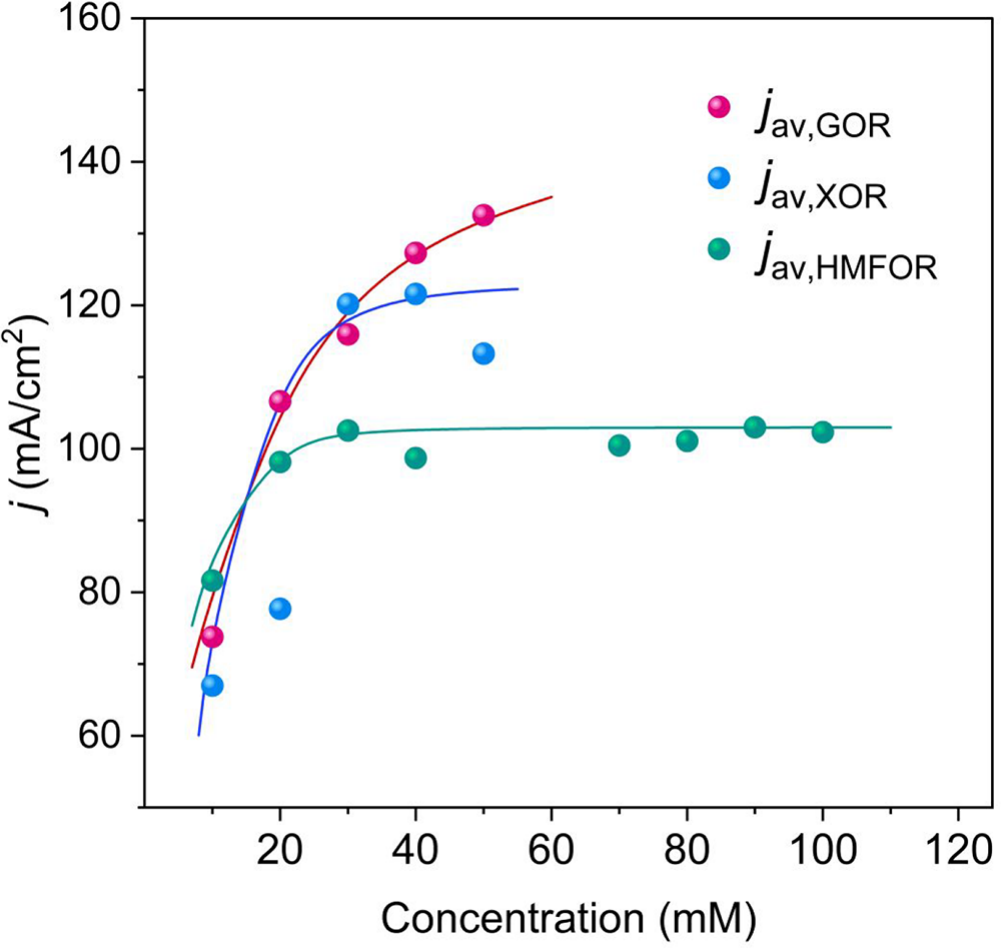
Average current densities (*j*_av_) obtained from 30 min chronoamperometry tests at different substrate concentrations for glucose (GOR), xylose (XOR), and HMF (HMFOR). Solid lines represent the corresponding modeled fits for each reaction.

At the molecular level, these trends can be rationalized by considering the direct competition between organic adsorbates and oxygenated intermediates on the NiOOH active sites. On activated Ni foam, Glc, Xyl, and HMF are oxidized via adsorption on NiOOH, where Ni^3+^ species act as the redox-active centers and are reduced to Ni^2+^ during organic oxidation. In parallel, the OER proceeds through the adsorbate evolution mechanism, involving the sequential formation of *OH, *O, and *OOH intermediates on the same surface sites. When organic surface coverage is sufficiently high, the continuous consumption of Ni^3+^ species kinetically hinders *OOH formation, leading to effective suppression of the OER. Conversely, when organic coverage decreases or surface poisoning occurs, *OH/*O intermediates dominate the surface, restoring OER while inhibiting organic oxidation.

Consistent with this interpretation, Rafaïdeen et al.^17^ reported that Xyl is chemically less stable than Glc and that increasing the concentration impairs the electrorefining performance by poisoning more rapidly the electrocatalysts than Glc, in addition to possible transport contributions associated with the different reaction intermediates and alkaline-driven side products from Xyl. In the case of HMF, however, a clear plateau is evident between 20 and 100 mM, with the current stabilized at 101.9 ± 2.1 mA. The persistence of this plateau reveals a dual limitation, combining mass transport and the intrinsic rate of HMFOR, such that increasing its concentration does not translate into a higher current. Notably, although the adsorption constant extracted for HMFOR modeling (*K* ≈ 160 M^–1^) is higher than those of Glc and Xyl, the overall steady yet lower current density reflects the combined impacts of intrinsic kinetic limitations, mass transport constraints, and inhibition phenomena, such as the accumulation of humins, factors which govern HMFOR as will be discussed further below. All this information has also been complementarily discussed in Figure S5 in the Supporting Information (pages S6 and S7).

Another similarity between XOR and GOR is that the OER onset at 10 mM Xyl also occurs at 1.49 V, and in both cases, the FE_O_2__ dropped to zero at 30 mM (Figure 4a). Nonetheless, in the literature, the inhibitory effect of XOR on the OER has been reported to a lesser extent than that of GOR, and the optimal concentration has not yet been systematically determined. Gan et al.^21^ reported a Ni–Co-based catalyst in a flow-cell for Xyl oxidation coupled with H_2_ production in 1.0 M KOH. Although FE_O_2__ was not reported, the suppression of OER was suggested by the EIS measurements (Bode plot), in which an additional process at ∼1.25 V was revealed, consistent with the changes in the Ni^3+^ and Co^3+^ redox state, upon the addition of 50 mM Xyl, while the typical OER signal (usually observed at ∼1.50 V) was attenuated up to 1.6 V, the highest potential examined. Similarly, Liu et al.^22^ using a Ni–Mo-based catalyst found by rotating ring-disk electrode analysis that, in 1.0 M KOH with 100 mM Xyl at 1.2 V, the current associated with *OH species increased in response to the presence of Xyl, suggesting a strong inhibitory effect on the OER as also observed at 1.6 V under the same conditions. In the present work, however, the evidence suggests that XOR dominates over the OER at 30 mM Xyl, lower than the concentrations reported in both studies, although it is less stable at higher concentrations.

**Figure 4:**
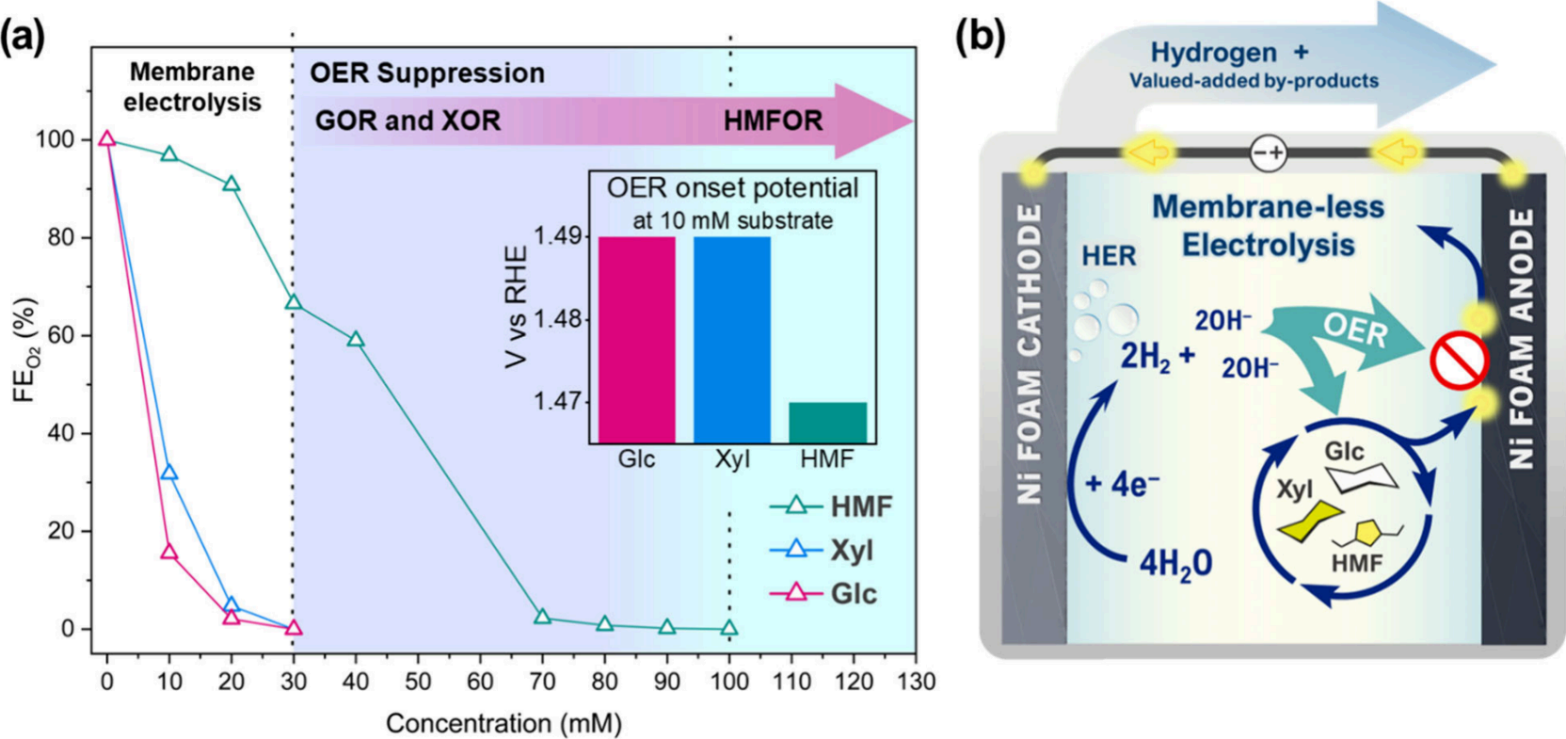
Proposed threshold concentrations enabling membraneless electrolysis. (a) Faradaic efficiency of O_2_ (FE_O_2__) as a function of substrate concentration, showing the suppression of the OER for glucose (Glc), xylose (Xyl), and HMF. (b) Schematic illustration of the membraneless electrocatalytic process for simultaneous H_2_ evolution and O_2_-free oxidation of biomass-derived molecules.

In contrast, the O_2_ faradaic efficiencies in the presence of HMF were significantly higher. In fact, the OER was suppressed only when the concentration of HMF reached 100 mM, as seen in Figure 4a. This figure clearly shows a summary of the OER competition study between the three substrates, establishing the operation region of a membraneless electrolysis process (Figure 4b). Unlike GOR and XOR, HMFOR is the most studied case for OER competition, whereas in Glc and Xyl, the more complex and less selective pathways relegate OER competition to a secondary role.^36,37^ Yet, no clear consensus exists on the HMF concentration required for effective OER suppression. A major factor lies in the narrow potential window for HMF oxidation. The literature consistently shows a near-linear correlation between HMFOR performance and the applied potential, especially ranging from 1.41 to 1.47 V.^20^ Beyond this range, the reaction becomes less efficient due to a dual effect: the oxidation of HMF eventually yields to the adsorption of OER intermediates on the active sites,^38,39^ while, independently of the applied potential, limited mass transport further constrains the process. Increasing the substrate concentration, provided that suitable transport conditions are maintained to minimize HMF degradation into humins, may mitigate these effects, sustaining FDCA selectivity and faradaic efficiency (FE_FDCA_).^38,40^ Otherwise, mass transport limitations inevitably promote HMF degradation and OER activity.

Nonetheless, although numerous reports highlight the apparent suppression of the OER, this is within the narrow potential window mentioned above. Moreover, the presumed absence of oxygen production is inferred only from indirect evidence: typically, LSV comparisons with and without HMF, EIS analysis, or FE_FDCA_ calculations only. Remarkably, the direct quantification of O_2_ remains almost entirely unaddressed in the literature. To the best of our knowledge, the only report is the study of Hauke et al.,^41^ where *operando* DEMS revealed no oxygen evolution up to ∼1.7 V in the presence of 10 mM HMF, providing direct evidence of genuine OER suppression. However, the authors themselves acknowledged that (1) these observations stem from transient microfluidic measurements at low HMF concentration and high electrolyte flow, which do not resemble steady-state operational conditions (such as in chronoamperometry), and (2) the OER may still be masked in the zero-gap MEA operated at 1.56 V, highlighting that the lack of oxygen detection cannot be definitively established without direct quantification.

While higher substrate oxidation currents can mask, though not fully suppress, the OER at the anode surface, in macroscopic systems, the competition between both reactions remains evident, such as in this study, where clear substrate-specific thresholds for OER suppression on NF are established from *in situ* O_2_ measurements. The distinct behavior of HMF compared to Glc and Xyl points to transport limitations and possible degradation into unrecoverable byproducts, such as humins,^20^ which is consistent with the reported literature. The faster adsorption rate estimated for HMF, together with the current plateau observed at relatively low concentrations (which starts at the same concentration at which the other two aldoses achieve OER suppression), indicates a rapid saturation of Ni active sites due to strong HMF adsorption. As the surface coverage (θs) approaches unity, the HMFOR is controlled by surface kinetics rather than reactant concentration. Moreover, the presence of humins, whose formation rate may be enhanced at increasing HMF concentrations, may further contribute to a lateral adsorption effect by acting as inert adsorbates that progressively block Ni active sites. Therefore, the behavior observed in Figure 3 reflects a combined effect suggesting an initial rate-limiting surface reaction step and the progressive inhibition of HMFOR due to site blocking. Future work should therefore focus on rigorous carbon balances and the implementation of suitable steady-state systems where suppression of the OER can be validated under realistic HMFOR operating conditions. Table S1 summarizes the main studies published in the literature using NF-supported catalysts, offering a perspective on the current state of OER suppression and its implications for improving HMFOR performance in NF-based systems. In comparison to other systems, the results of the present study with *in situ* O_2_ monitoring fall within the average range of HMF concentration where OER suppression has been observed.

In summary, Glc showed the lowest FE_O_2__ values with the broadest window for membraneless operation, better than Xyl, as seen by the average current at concentrations above 30 mM. In contrast, due to kinetic limitations and the strong competition for active sites by *OH species, HMF appears to contribute less to sustaining the membraneless operating regime, thus requiring higher concentrations to achieve full suppression of the OER. Therefore, Glc was selected to carry out an extended CA test at 1.6 V, accompanied by HPLC analysis to evaluate the viability of a membraneless process. Moreover, as a model molecule, the end-product analysis allowed direct comparison with recent literature reports on Glc electro-oxidation over Ni-based catalysts.

### 3.4 Long-Term GOR Chronoamperometry and HPLC Analysis

Following the previous screening study and considering Glc as the model molecule, a long-term CA experiment at 1.6 V vs RHE with 100 mM Glc was carried out for 15 h, with HPLC analysis performed each hour up to the point at which the OER began to compete. The selection of this operation potential was based on ensuring strong organic oxidation while retaining a reasonable competition between the OER. Hence, this potential is sufficiently anodic to activate NiOOH and drive the oxidation of all substrates (as supported by our previous experiments) while avoiding unnecessarily extreme potentials that could lead to side reactions or material degradation. The main intermediates and end products identified by HPLC were formic (FA), oxalic (OA), lactic (LA), and glycolic (GlycA) acids. These compounds, together with additional products whose presence is discussed further below, had already been unambiguously identified by NMR in our previous GOR–OER competition study,^15^ thereby supporting the reliability of the present qualitative peak assignments (Figure 5b,c). In the first place, from Figure 5a it can be clearly observed that the time window in which the system can operate free of O_2_ at 1.6 V is around 4 h (MLE operation time window). During this period of time, no oxygen was detected, and no gas bubbles were observed in the anode compartment of the cell. At *t* = 4 h, the Glc concentration determined was 33.5 mM, and the OER began to compete right (O_2_ production was detected), which is in good agreement with the experiments shown in Figure 4a. Since the HPLC chromatograms indicate formate as the major product, faradaic efficiency calculations toward formate were performed. The faradaic efficiency was approximately 55% during the first hour, followed by a slight decrease and stabilization at around 50% up to 4 h (Figure S6). In addition, glucose conversion analysis revealed a conversion of approximately 70% within the 4 h operation window (Figure S6).

**Figure 5:**
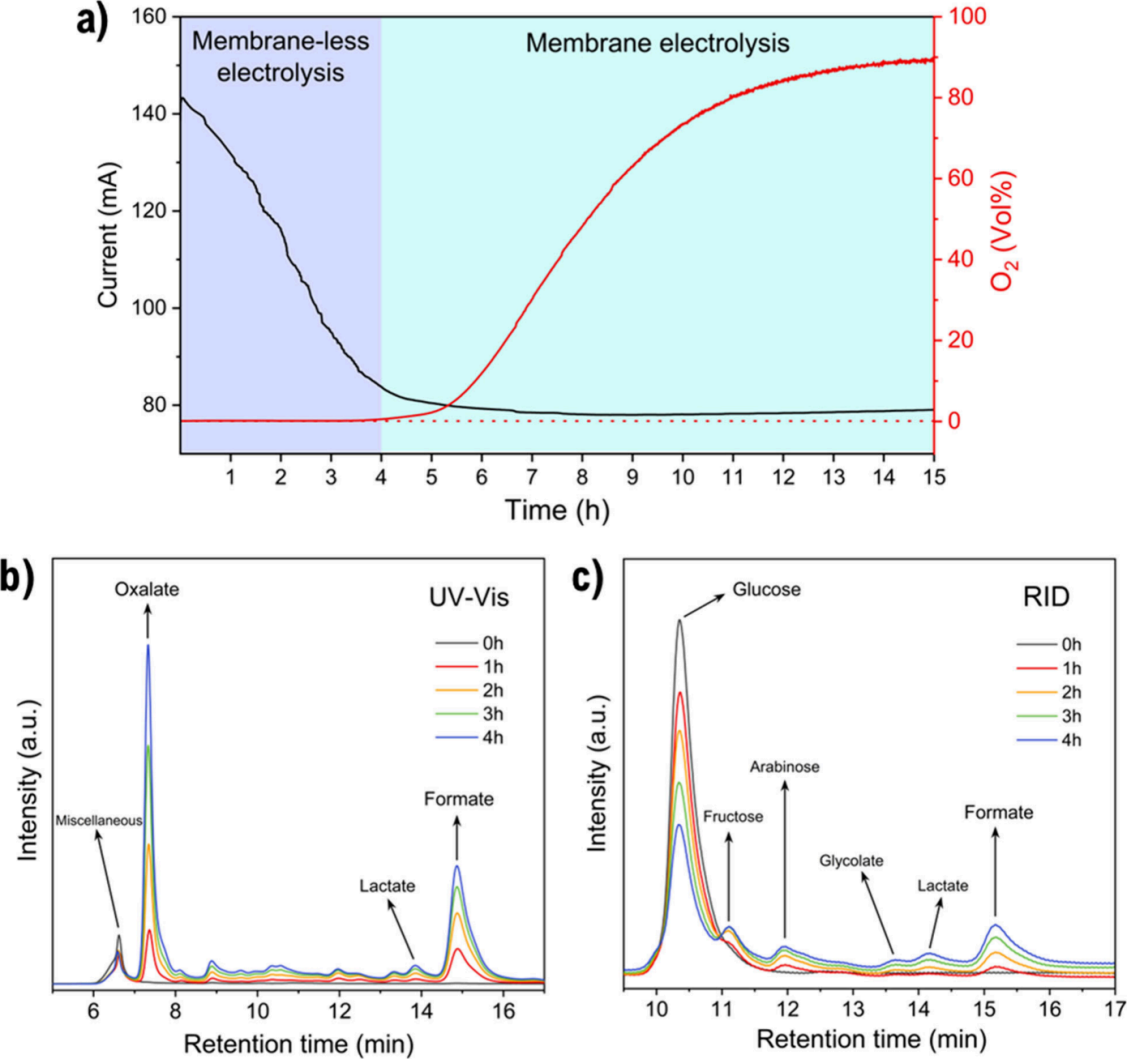
Long-term glucose oxidation. (a) Chronoamperometry at 1.6 V (15 h) under membraneless electrolysis conditions, showing an initial 4 h period without O_2_ evolution. HPLC chromatograms with (b) UV–vis detection at 210 nm and (c) reflective index (RID), indicating the byproducts identified after 4 h of electrolysis.

The electrochemical and nonelectrochemical pathways of Glc oxidation are an intricate topic that continues under intense research.^29,32^ In the literature, it is generally agreed that gluconolactone is a primary intermediate from Glc electro-oxidation, from which gluconic acid (GA) is derived.^47,48^ Nevertheless, in our analysis, the peak associated with GA was too weak to be clearly identified, which is consistent with previous reports where GA was scarcely detected.^49^ Another reaction pathway recently proposed involves oxidative C–C cleavage. At potentials above 1.2 V, Glc is adsorbed on the Ni surface, and the resulting intermediate undergoes cleavage of the C1–C2 bond, yielding formate and a C5 fragment. Upon desorption, Ni^2+^ is regenerated, while the released Glc-derived species is deprotonated by OH^–^ in solution, leading to the formation of arabinose (Ara).^29,31^ This proposed mechanism explains not only the promotion of FA but also the accumulation of Ara, as seen in Figure 5c. In turn, OA is produced through several pathways, mainly through the electrochemical oxidation of GOR-derived GlycA and tartronate.^50^ In this work, however, the OA concentration after 4 h attained roughly 2–3 mM. In contrast, the FA concentration determined at the same time was 103 mM, emerging as the main end product of this process, formed mainly through the above-mentioned oxidative C–C cleavage pathway, since the FA from alkaline-driven nonelectrochemical reactions is negligible, confirmed by the HPLC of a 4 h test without applied voltage.

It is worth noting that, in contrast to conventional industrial routes for formic acid production such as CO-based carbonylation processes operated under high pressure (Kemira–Leonard process),^51^ GOR enables formate formation under mild electrochemical conditions using a renewable feedstock. Operating at ∼1.6 V vs RHE, this approach allows the suppression of the OER while simultaneously producing formate as a value-added coproduct. As such, GOR represents a promising CO-free pathway that complements established thermochemical methods and highlights the potential of biomass-assisted electrolysis systems. Emerging as the main end product in our system, it is relevant to also briefly address formate downstream separation. First, an optional step could be the removal of the residual oxalate formed, which can be selectively removed by precipitation with Ca^2+^ as calcium oxalate, a classical approach that has been widely studied and is known for its robustness and well-established kinetics.^52^ Then, as formate remains highly soluble under alkaline conditions, its purification will rely mainly on membrane-based technologies, which are currently the most applied and economically relevant route for large-scale recovery.^53^ An alternative pathway of increasing interest is the formate-to-oxalate coupling reaction (FOCR), where formate is converted to oxalate under thermal or catalytic conditions. These reactions, first described more than a century ago, have gained renewed attention in recent years as part of the CO_2_ utilization framework.^54^

## 4 Conclusions

In this study, the competition of the OER vs GOR, XOR, and HMFOR was systematically assessed under alkaline conditions (1.0 M NaOH), by coupling chronoamperometry with real-time *in situ* optical O_2_ sensing. An activated NF was used as the anode. First, a multipotential step approach at a substrate concentration of 10 mM showed an OER onset potential of 1.49 V for Glc and Xyl and 1.47 V for HMF. At 1.6 V, full OER suppression was achieved at 30 mM for Glc and Xyl. In contrast, HMF exhibited stronger kinetic limitations, and OER was not fully suppressed until 100 mM. These results are consistent with the narrower potential window and transport constraints of HMFOR. Being the most interesting compound for a membraneless process, Glc was selected to conduct a long-term operation given its stability and favorable kinetics. A practical 4 h membraneless operation window was found before OER progressively dominates, which coincides with the decline of Glc concentration around 30 mM after this period. HPLC analysis also revealed a high selectivity toward FA. Overall, these findings demonstrate the feasibility of simultaneously producing H_2_ and value-added chemicals in a membraneless electrolyzer. This work provides practical insights into OER suppression that could support the development of simpler and cost-effective systems for sustainable H_2_ and chemical production from biomass.

## Supplementary Material



## Data Availability

The data that support the findings of this study are available on Zenodo: 10.5281/zenodo.18241483.

## Notes

EDX mapping, XPS spectra of Ni foam before and after activation, electrochemical oxidation studies of xylose and HMF, fitting curves of oxidation currents, FE toward formate and glucose conversion, and comparison of OER suppression in HMFOR with the literature (PDF)
