# Supplementary material for: Activated Nickel Foam Anodes for Sustainable Biomass Valorization: Competitive Oxidation of Organic Molecules vs the Oxygen Evolution
Source: Energy Fuels. 2026 Feb 16;40(8):4277–87. doi: 10.1021/acs.energyfuels.5c05778 (PMC12951432; doi:10.1021/acs.energyfuels.5c05778)
Supplement: Supplementary file 1 [file ef5c05778_si_001.pdf]

Supporting Information

Activated Nickel Foam Anodes for Sustainable Biomass Valorization: competitive oxidation of organic molecules vs the oxygen evolution

Rudy Crisafulli[2] †, I. Rafael Garduño-Ibarra [1] †, Sravan K. Kilaparthi[1] †, Paula Sánchez[1], Antonio de Lucas-Consuegra[1]*

[1] Department of Chemical Engineering, Faculty of Chemical Sciences and Technologies, University of Castilla-La Mancha, Ciudad Real, 13005, Spain

[2] Instituto de Pesquisas Energéticas e Nucleares, IPEN/CNEN-SP, Av. Professor Lineu Prestes 2242, CEP 05508-000, São Paulo-SP, Brazil

† *These three authors contributed equally to this work*

Corresponding authors: * Antonio.Lconsuegra@uclm.es

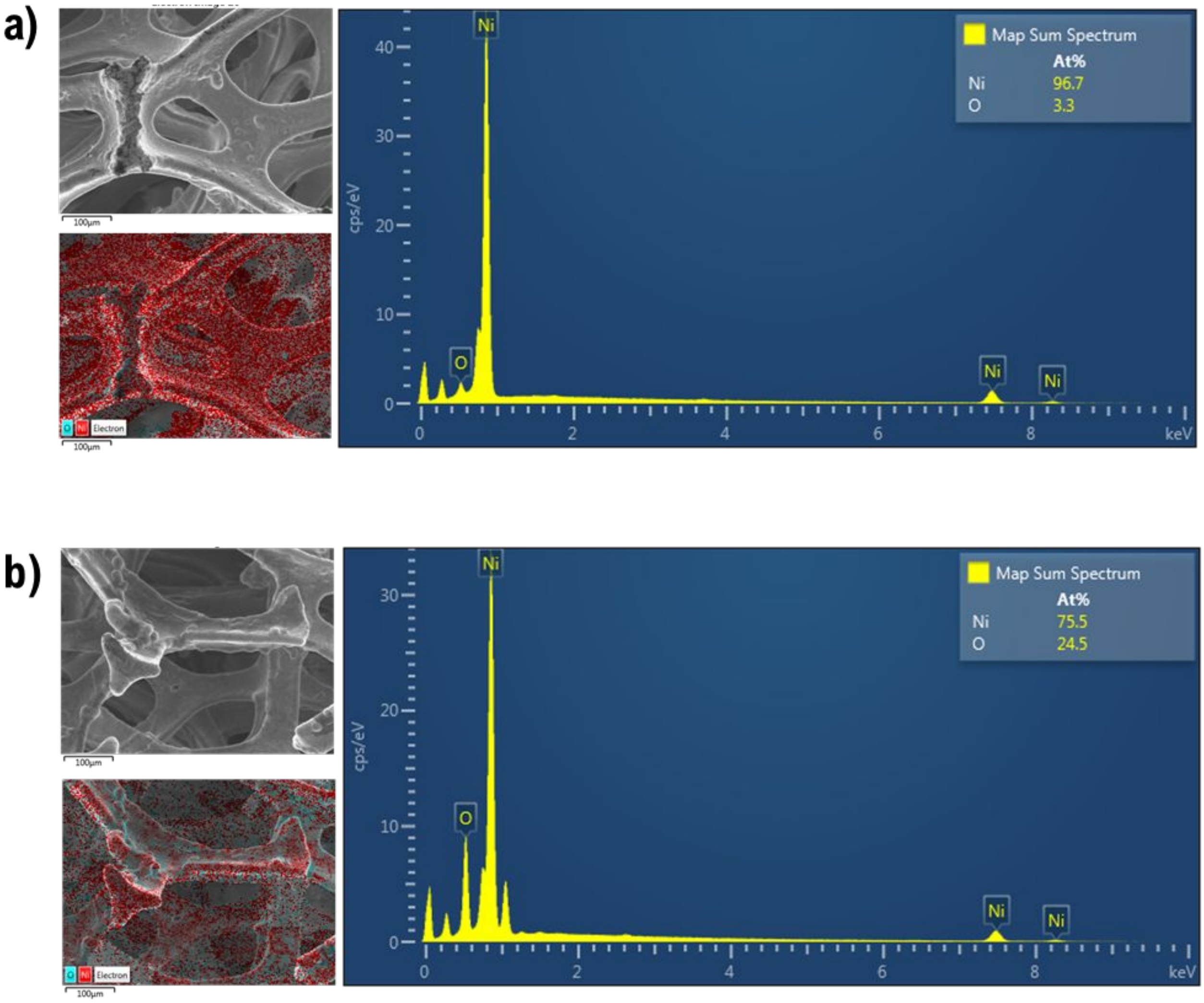

Figure S1. EDX mapping: a) bare Ni foam and b) Ni foam after activation

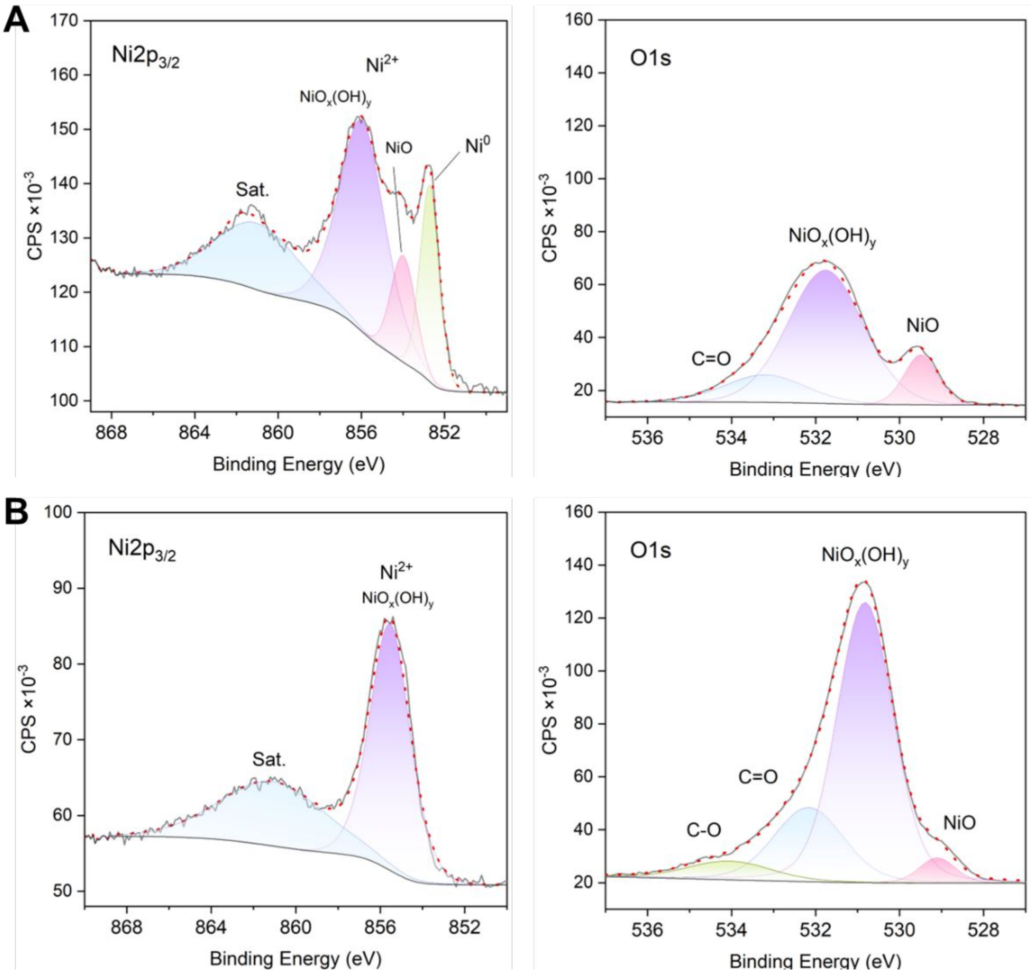

Figure S2. XPS of A - Ni foam before activation; B - Ni foam after activation.

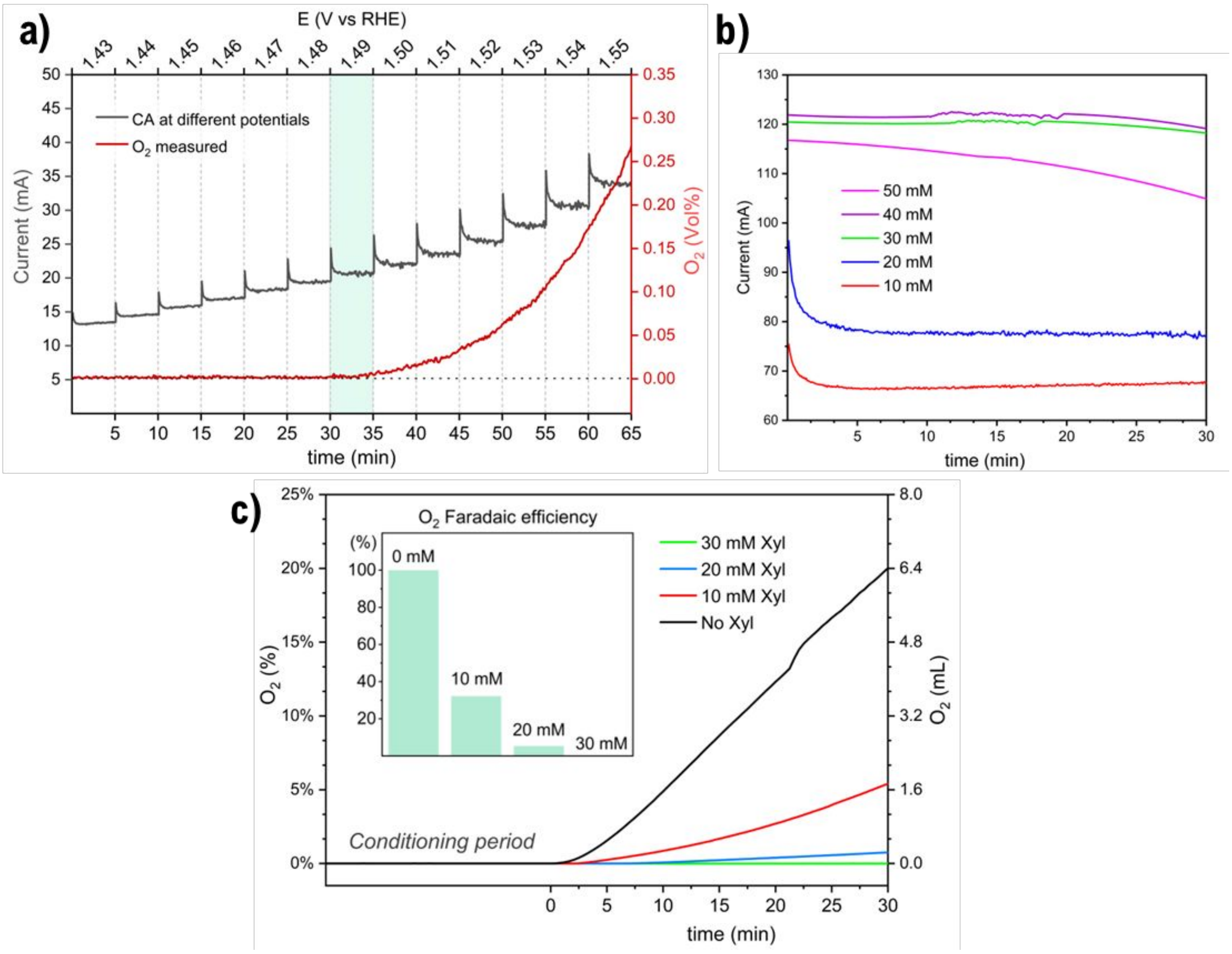

Figure S3. Xylose (Xyl) electro-oxidation in alkaline medium (1.0 M NaOH) on activated Ni foam. a) Multi-step chronoamperometry with *in-situ* O_2 detection at 10 mM Xyl to determine the O_2 onset potential. b) Chronoamperometric profiles at different Xyl concentrations (30 min). c) O_2 evolution during the 30 min tests and the corresponding faradaic efficiency of O_2 (FE_{O_2}) showing clear OER suppression at 30 mM Xyl.

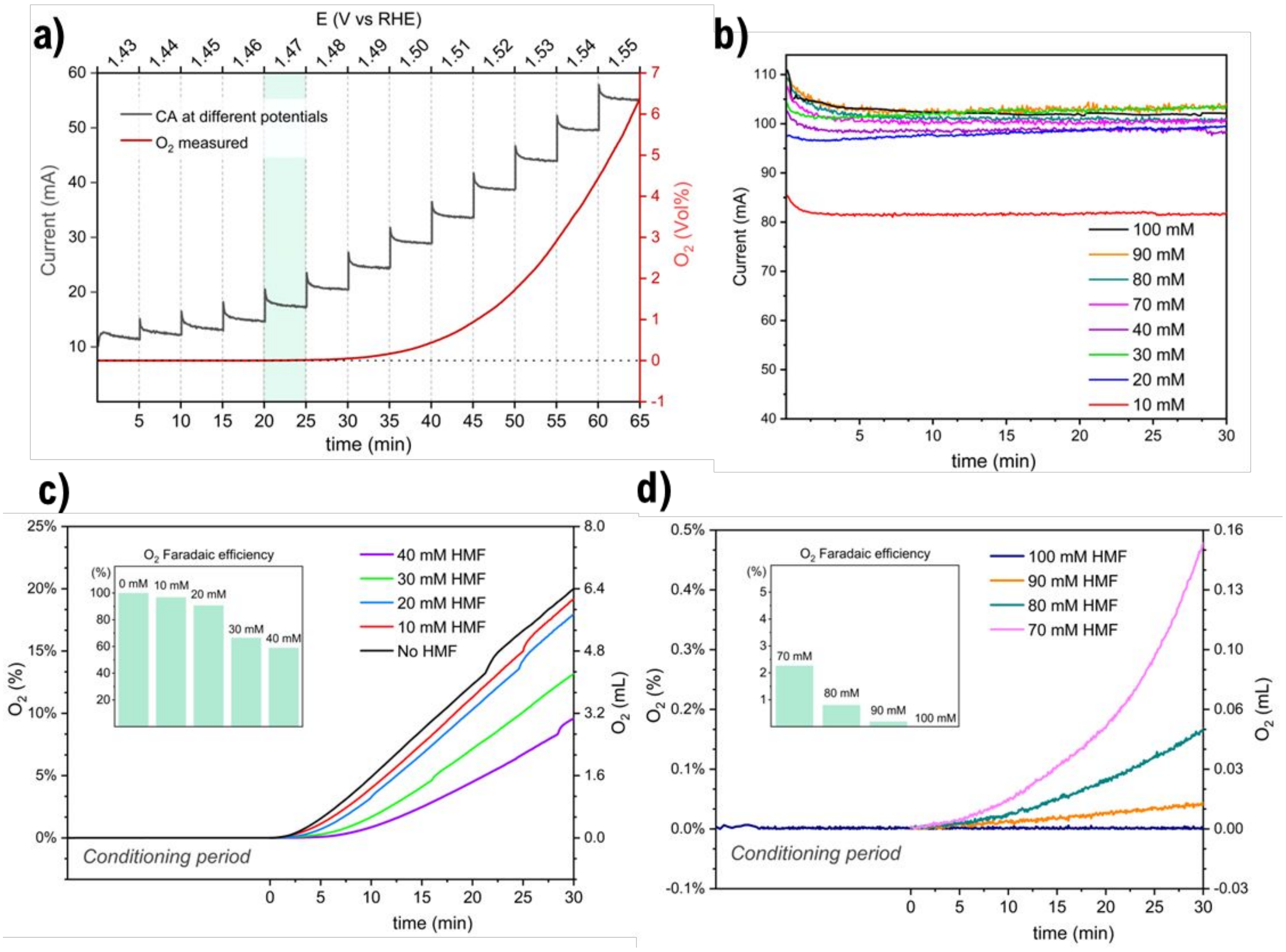

Figure S4. 5-hydroxymethylfurfural (HMF) electro-oxidation in alkaline medium (1.0 M NaOH) on activated Ni foam. a) Multi-step chronoamperometry with *in-situ* O_2 detection at 10 mM HMF to determine the O_2 onset potential. b) Chronoamperometric profiles at different HMF concentrations (30 min). c) and d) O_2 evolution during the 30 min tests and the corresponding faradaic efficiency of O_2 (FE_{O_2}) showing clear OER suppression at 100 mM HMF.

Relationship between substrate concentration and current density used to characterize the co-electrolysis of water and organic molecules

In the co-electrolysis of water and organic substrates, the electrochemical current is associated with a potential-activated elementary step, typically involved in oxygen evolution or organic electro-oxidation, which is based on the Butler-Volmer kinetics for an irreversible reaction:

$$i = FAk^0 \exp\left[\frac{\alpha F\left(E - E_i^0\right)}{RT}\right] C_{OH^-}$$

where i is the current, F the Faraday constant, A the electrode area, k^0 the rate constant, α the transfer coefficient, and R and T the gas constant and temperature. C_{OH^-} denotes the OH^- concentration. The backward electrochemical step is neglected given the strong irreversibility of the oxygen evolution reaction. In the absence of organic compounds, the current increases with OH* surface coverage (θ_{OH*}). The reaction rate between OH* and the adsorbed substrate is:

$$r = k \cdot \theta_{OH*} \cdot \theta_{sub*}$$

Where r is the reaction rate, k is the reaction rate constant, θ_{OH*} and θ_{sub*} represent the surface coverages of OH* and organic substrate. The reaction rate follows the reaction rate for a bimolecular adsorption reaction via a Langmuir–Hinshelwood (L–H) mechanism at gas-solid interfaces:

$$r = k \cdot \theta_A \cdot \theta_B$$

In the simultaneous electro-oxidation of water and organic compounds, the reaction is positively correlated with the electrochemical current. The reaction rate equation can be also transformed into a function of θ_{sub*}:

$$\theta_{OH*} = 1 - \theta_{sub*}$$

$$r = k \cdot \theta_{sub*}^2 + k \cdot \theta_{sub*}$$

Within a certain concentration range θ_{sub*} becomes proportional to $\log(C_{sub})$ resembling the classical Temkin isotherm where $\theta \propto \log(C)$ for monolayer chemisorption. Therefore, the reaction rate equation can be expressed as follows:

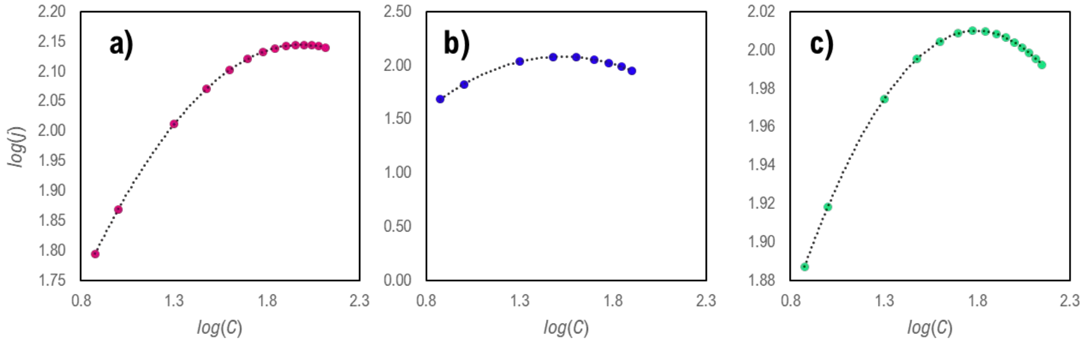

$$r = k' \cdot \log(C_{sub})^2 + k' \cdot \log(C_{sub})$$

Figure S5. The fitted parabolic curve for electrochemical oxidation currents. a) glucose; b) xylose; and c) HMF

With this approach, it was possible to estimate the maximum reaction rate and to predict the positive correlation between reaction rate and electrochemical current density (**Figure S4**). For each compound, from the θ_{sub*} at the max. reaction rate, the corresponding estimated concentration was 100 mM, 40 mM and 60 mM, for glucose, xylose and HMF, respectively. This approach presented by Tian et al.[1], enables to predict information which was used to fit the current as a function of the concentration of **Figure 3** in the text.

The surface coverage of the adsorbed OER (θ_{OH*}) and organic species (θ_{sub*}) is given by the Langmuir adsorption isotherm:

$$\theta_{OH*} = \frac{1}{(1 + KC)} \qquad \theta_{sub*} = \frac{KC}{(1 + KC)}$$

In a simplified L-H model approach, the rate is expected to vary with the surface site density, *i.e.*, $r \propto [*]_o{}^m$, where m is the number of simultaneously empty surface sites that the electrode must present for the rate-determining step of the OER to proceed[2]:

$$\theta_{OH*'} = \frac{1}{(1 + KC)^m} \qquad \theta_{sub,eff*} = \frac{KC^n}{(1 + KC)^n}$$

If the OER requires a neighboring pair of sites (*e.g.*, M–O* / M–OH*), m is expected to be 1 or 2, but if OER requires a larger ensemble, then m can be > 2. Given the competition in the presence of organic compounds, in this study it was explored m > 2. On the other hand, a Hill–Langmuir occupancy with exponent n was adopted as a compact, physically motivated proxy for surface heterogeneity and multi-site cooperativity in the case of substrate coverage $\theta_{sub,eff*}$. This approach has been used in adsorption/catalysis with modern multi-ensemble micro-kinetics[3].

Finally, the predicted total current is calculated from:

$$j_{tot}(C) = j_{OER}(C) + j_{sub,max}(C) = \frac{1}{(1 + KC)^m} + \frac{KC^n}{(1 + KC)^n} \times k \cdot C_{eff}$$

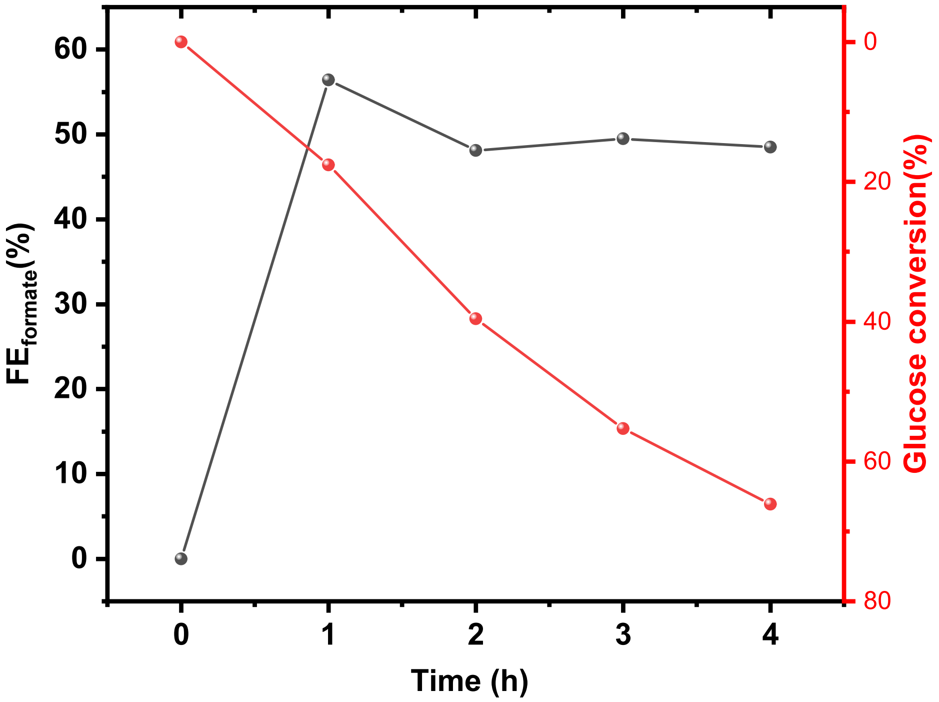

Figure S6. Faradaic efficiencies towards formate and glucose conversion (%) during the CA experiment (until 4h) derived from the HPLC chromatograms.

Table S1. OER suppression in HMFOR using Ni foam-supported catalysts

Configuration	Catalyst	Support	Electrolyte	[HMF] (mM)	E (V vs. RHE)	OER suppression	O_2 measured	Method	Ref
H-cell	Activated NF	—	1.0 M KOH	100	1.60	Yes	Yes	O_2 sensor FE_{O_2}	This study
3E-op cell	NiV LDH	NF	0.1 M KOH	5 / 100	1.45	inferred	No	operando XAS/EXAFS	4
3E-op cell / Zero-gap MEA	NiFe-LDH/Cl⁻	NF	0.1 M KOH	10	< 1.70 / ≤ 1.56	Yes	Yes	DEMS FE_{FDCA}	5
3E-op cell / H-cell	$MoNi_4$	NF	0.1 M KOH / 1.0 M KOH	10 / 200	1.45	inferred	No	*in-situ* Raman, FE_{FDCA}	6
H-cell	NiB@NF	NF	1.0 M KOH	10	≤ 1.50	inferred	No	~100% FE_{FDCA}	7
H-cell	d-NiMnFe-LDH	NF	1.0 M KOH	50	1.50	slowed	No	EIS, FTIR in-situ, LSV	8
2E-op CSTR	NiFeOOH	NF	0.3 M KOH	100	1.80	partial	No	FE_{FDCA}	9
H-cell	a-$Ni(OH)_2$–Cu_2O	NF	1.0 M KOH	100	1.42	inferred	No	LSV, FE_{FDCA}	10

3E-op - three-electrode operation; 2E-op - two-electrode operation; CSTR – continuously stirred tank reactor

References

(1) Tian, B.; Wang, F.; Ran, P.; Dai, L.; Lv, Y.; Sun, Y.; Mu, Z.; Sun, Y.; Tang, L.; Goddard Iii, W. A.; Ding, M. *Supplementary Information for Parameterization and Quantification of Two Key Operando Physio-Chemical Descriptors for Water-Assisted Electro-Catalytic Organics Oxidation*.

(2) Kiani, D.; Wachs, I. E. The Conundrum of "Pair Sites" in Langmuir-Hinshelwood Reaction Kinetics in Heterogeneous Catalysis. *ACS Catalysis* **2024**, *14* (13), 10260–10270. https://doi.org/10.1021/acscatal.4c02813.

(3) Khoshraftar, Z.; Ghaemi, A.; Hemmati, A. Comprehensive Investigation of Isotherm, RSM, and ANN Modeling of CO2 Capture by Multi-Walled Carbon Nanotube. *Sci Rep* **2024**, *14* (1). https://doi.org/10.1038/s41598-024-55836-6.

(4) Kim, K. H.; Jang, H.; Woo, J.; Lee, M. Y.; Kim, M. G.; Moon, B. C.; Lee, D. K. NiV Layered Double Hydroxide for Efficient and Scalable Electrochemical Oxidation of 5-Hydroxymethylfurfural to High-Purity 2,5-Furandicarboxylic Acid. *ACS Catal* **2025**, *15*, 14693–14702. https://doi.org/10.1021/acscatal.5c03589.

(5) Hauke, P.; Klingenhof, M.; Wang, X.; de Araújo, J. F.; Strasser, P. Efficient Electrolysis of 5-Hydroxymethylfurfural to the Biopolymer-Precursor Furandicarboxylic Acid in a Zero-Gap MEA-Type Electrolyzer. *Cell Rep Phys Sci* **2021**, *2* (12). https://doi.org/10.1016/j.xcrp.2021.100650.

(6) Liu, H.; Xia, J.; Liu, X.; Hu, Y.; Shakouri, M.; Wu, H.; Zhu, M.; Guo, Y.; Chen, J.; Wang, H.; Wang, Y. Bifunctional MoNi4/Nickel Foam Electro-Catalyst for Ultra-Efficient Oxidation of High-Concentration 5-Hydroxymethylfurfural and HER. *ChemSusChem* **2025**, *18* (5). https://doi.org/10.1002/cssc.202401516.

(7) Barwe, S.; Weidner, J.; Cychy, S.; Morales, D. M.; Dieckhöfer, S.; Hiltrop, D.; Masa, J.; Muhler, M.; Schuhmann, W. Electrocatalytic Oxidation of 5-(Hydroxymethyl)Furfural Using High-Surface-Area Nickel Boride. *Angewandte Chemie - International Edition* **2018**, *57* (35). https://doi.org/10.1002/anie.201806298.

(8) Zheng, H.; Wang, X.; Shi, K.; Hu, Y.; Lv, W.; Yin, P.; li, C.; Sun, L.; Wang, J.; Shen, B.; Liu, H. Cationic Defect Engineering for Promoting Oxidation of 5-Hydroxymethylfurfural While Passivating OER. *ACS Appl Mater Interfaces* **2025**. https://doi.org/10.1021/acsami.5c12891.

(9) Chakthranont, P.; Woraphutthaporn, S.; Sanpitakseree, C.; Srisawad, K.; Faungnawakij, K. Kilogram-Scale Production of High Purity 2,5-Furandicarboxylic Acid via Sustainable Leap in Continuous Electrochemical Oxidation of 5-Hydroxymethylfurfural. *Chemical Engineering Journal* **2023**, *476*. https://doi.org/10.1016/j.cej.2023.146478.

(10) Qi, Y. F.; Wang, K. Y.; Zhou, Y.; Sun, Y.; Wang, C. Effects of Different Vacancies in Nickel Hydroxides on the Electrooxidation towards 5-Hydroxymethylfurfural. *Chemical Engineering Journal* **2023**, *477*. https://doi.org/10.1016/j.cej.2023.146917.
